# Clinical Impact of after-consult clinic blood pressure: comparison with automated office blood pressure

**DOI:** 10.1186/s40885-021-00171-5

**Published:** 2021-08-01

**Authors:** Cheol Ho Lee, Ji Hun Ahn, Joon Ha Ryu, Woong Gil Choi

**Affiliations:** 1 Cardiology, Konkuk University Chungju Hospital, Chungju, South Korea; 2 Internal medicine, School of Medicine, Konkuk University, 82, Gugwon-dearo, Chungju, Chungbuk 27376 Chungju, South Korea

**Keywords:** Blood pressure, Whitecoat, Hypertension

## Abstract

**Background:**

It is most important to measure blood pressure (BP) exactly in treating hypertension. Recent recommendations for diagnosing hypertension clearly acknowledge that an increase in BP attributable to the “whitecoat response” is frequently associated with manual BP recordings performed in community-based practice.

However, there was no data about after-consult (AC) BP that could reduce whitecoat effect. So we evaluated before-consult (BC) and AC routine clinic BP and research based automated office blood pressure (AOBP) measured.

**Methods:**

The study population consisted of 82 consecutive patients with hypertension between April 2019 and December 2019. We measured routine clinic BP and AOBP before and after see a doctor, respectively. Seated blood pressure and pulse are measured at each time after a rest period using an automated device as it offers reduced potential for observer biases. AOBP was measured and measuring BP 3 times un-observed. We compared each BP parameter for identifying exact resting BP state.

**Results:**

There was significant difference between BC and AC systolic BP (135.37 ± 16.90 vs. 131.95 ± 16.40 mmHg, *p* = 0.015). However there was no difference in the BC and AC diastolic blood pressure (73.75 ± 11.85 vs. 74.42 ± 11.71 mmHg, *p* = 0.415). In the AOBP comparison, there was also significant difference (BC systolic AOBP vs. AC systolic AOBP, 125.17 ± 14.41 vs. 122.98 ± 14.09 mmHg, *p* = 0.006; BC diastolic ABOB vs. AC diastolic AOBP, 71.99 ± 10.49 vs. 70.99 ± 9.83, *p *= 0.038).

**Conclusions:**

In our study, AC AOBP was most lowest representing resting state. Although AC BP was higher than BC AOBP, it might be used as alternative measurement for reducing whitecoat effect in the routine clinical practice.

## Background

The effect of blood pressure (BP) to cardiovascular disease is obvious [1]. It has been well known that early intensive BP control affect benefit that reduced long term mortality or target organ damages [2]. Therefore, it is important to recognize and minimize early vascular and organ damages.

There is no concern that method of BP measurement is basic and important step in the hypertensive patient management. Hypertension guidelines described precisely BP measurement dividing conventional office BP, unattended office BP, out of office BP, Home BP, and ambulatory BP [3, 4]. Generally, values of measured BP in the doctor’s office is still cornerstone of BP control and hypertension treatment and BP is usually measured before see a doctor.

However, an increase in BP attributable to the “white coat response” is frequently associated with manual office BP recordings performed in community-based practice [5–7]. So, we designed after-consult (AC) BP measurement. This would be reflected more comfortable, physically stable state of patient. However, there was no study whether AC BP is less variable and could reflect long-term prognosis or not.

We evaluated before-consult (BC) and AC clinic BP and research based automated office blood pressure (AOBP) measured.

## Methods

### Study population

Participants were required to meet all the following criteria: controlled hypertensive state with medication, older than 18 years, no medication change during recent 3 month. Detailed exclusion criteria are one or more of following below: patients with secondary hypertension, uncontrolled diabetes mellitus, chronic kidney disease needing dialysis, more 2 times of AST/ALT, drug sensitivity, atrial fibrillation or uncontrolled arrhythmia. All participants provided written informed consent.

All major classes of antihypertensive agents were included in the formulary and we did not change medications during follow up period as possible as. Study investigators could also prescribe other antihypertensive medications.

### Blood pressure measurement

We measured three BP: office BP, AOBP and 24 h ambulatory BP (ABP). For office BP, an initial single observed BP measurement with non-invasive oscillometric system (OMRON HBP 1300, Japan) was performed after 5 min of quiet rest period using appropriate cuff size for mid-arm circumference, seated in chair, cuff at mid sternal level, arm supported on a flat surface, feet flat on the floor, with no conversation during the measurement.

AOBP was performed with another device (OMRON HEM-907XL, Japan) with the technique noted above but with the patient entirely alone in the exam room resting quietly and mean of three blood-pressure measurements was reported.

Twenty-four-hour ABP was monitored using validated oscillometric arm devices (TM-2430, A&D Company, Tokyo, Japan). Measurements were performed at 15–30 min intervals for 24 h, and study participants were instructed to remain still with the forearm extended during each BP reading. Awake and night-time periods were defined 07:00 to 22:00, 22:01 to 06:59, repetitively. ABP recordings with less than 70 % usable BP readings were excluded. All valid awake and nighttime ABP readings were averaged to provide a single awake and nighttime ABP value per study participant.

**Fig. 1:**
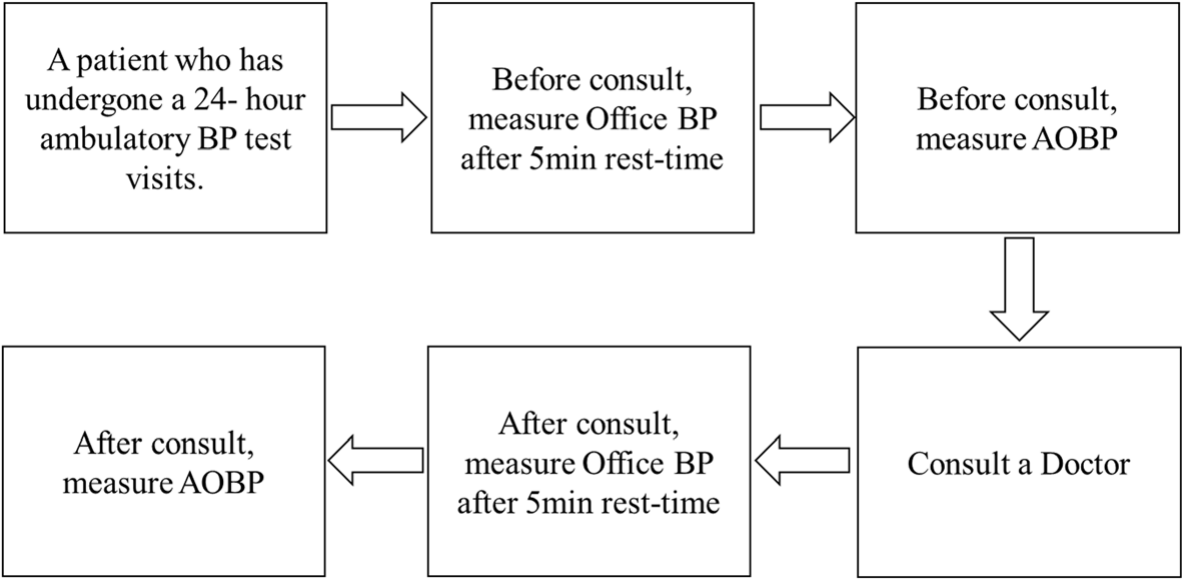
BP measurement pathway. BP: blood pressure

For office BP and AOBP, we repeated BP measurement after and before see a doctor with same method. We illustrated BP measurement pathway (Fig. 1).

For identifying target organ damage, we evaluated left ventricular hypertrophy, pulse wave velocity and microalbuminuria. Left ventricular hypertrophy (LVH) was defined by the increased left ventricular mass index (LVMI) in transthoracic echocardiography. (LVMI > 115 g/m2 in men and LVMI > 95 g/m2 in women), Pulse Wave Velocity (PWV) was measured by pulse waveform analyzer. (Vp-1000 plus, Omron, Japan), Urine Albumin-to-creatinine ratio (A/C ratio) was measured by a spot urine sample.

### Statistics

Continuous variables are reported as mean ± standard deviation (SD). Frequencies are given as percentages. Differences between mean systolic and diastolic AOBP values and heart rate (HR) were assessed using paired t tests for “white coat effect” measurements. Similarly, differences between mean systolic and diastolic AOBP and 24-hour BP values were also assessed using t tests. We compared agreement between BP measurements in two ways: We used the method of Bland and Altman with bias (defined as the mean value of the differences) and 95 % limits of agreement with their confidence intervals. The analyses were performed using the software R, version 3.2.2 (R Foundation for Statistical Computing, Vienna, Austria).

## Results

A total of eighty two consecutive hypertensives fulfilling the criteria for enrollment were included in the study, 46 men and 36 women, with a mean age of 62.6 ± 13.7 years. Their clinical characteristics are shown in Tables 1 and 2.

**Table 1:** Clinical characteristics

Variables	*N* = 82 pts (%)
Age, year	62.6 ± 13.7
Sex, male	46 (43.9)
Dyslipidemia	43 (52.4)
Chronic kidney disease	11 (13.4)
Diabetes mellitus	21 (25.9)
Heart failure	4 (5.4)
Stroke	6 (7.3)
Myocardial infarction	6 (7.3)
Hemoglobin, g/dL	13.87 ± 1.62
Platelet	245.8 ± 69.1
BUN, mg/dL	15.7 ± 5.0
Creatinine, mg/dL	1.0 ± 1.1
HemoglobinA1c, %	6.2 ± 0.9
hs-CRP, mg/L	1.0 ± 1.1
Total cholesterol, mg/dL	161.6 ± 39.0
Triglyceride, mg/dL	134.9 ± 83.7
LDL-cholesterol, mg/dL	86.2 ± 35.0
A/C ratio	48.2 ± 99.5

*BUN* Blood urea nitrogen, *hs-CRP* high sensitivity C reactive protein, *LDL-cholesterol* Low density lipoprotein cholesterol, *A/C ratio* Albumin to creatinine ratio

**Table 2:** Medication

Variables	*N* = 82 pts (%)
Antiplatelet	39 (47.6)
Numbers of BP medication	2.27 ± 0.96
Calcium channel antagonist	50 (64.1)
ARB	64 (81.0)
ACE inhibitor	1 (1.3)
Beta blocker	31(37.8)
Diuretics	12 (15.2)
Vasodilator	3 (3.8)
Alpha blocker	7 (9.3)

*BP* Blood pressure, *ARB* Angiotensin receptor blocker, *ACE* Angiotensin converting enzyme

**Fig. 2:**
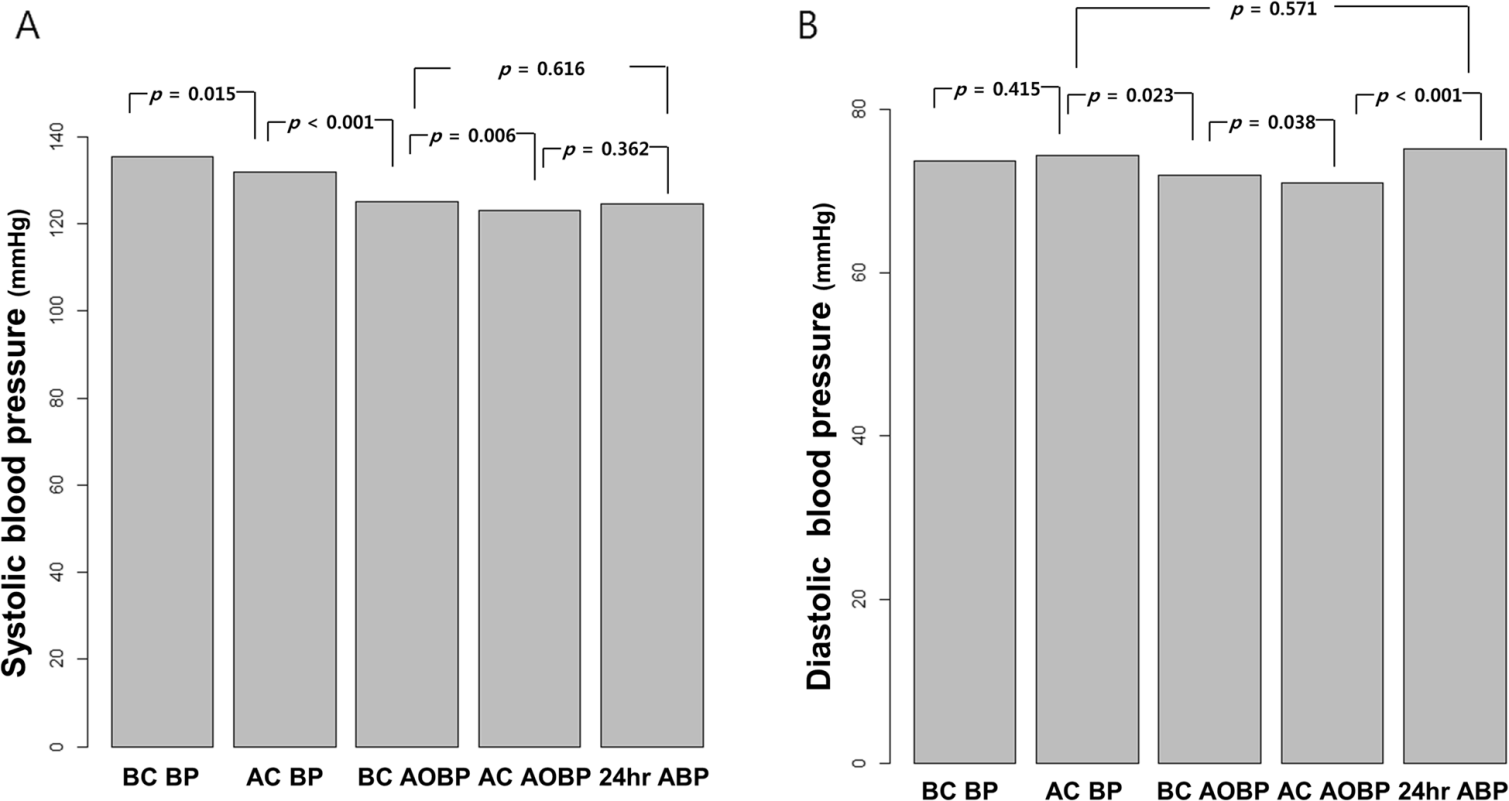
Comparison among office, automated blood pressure, and 24 h ambulatory blood pressure. Systolic BP comparison (**A**), Diastolic BP comparison (**B**). BC; before consult, BP; blood pressure, AC; after consult, AOBP; automated office blood pressure, ABP; ambulatory blood pressure

**Fig. 3:**
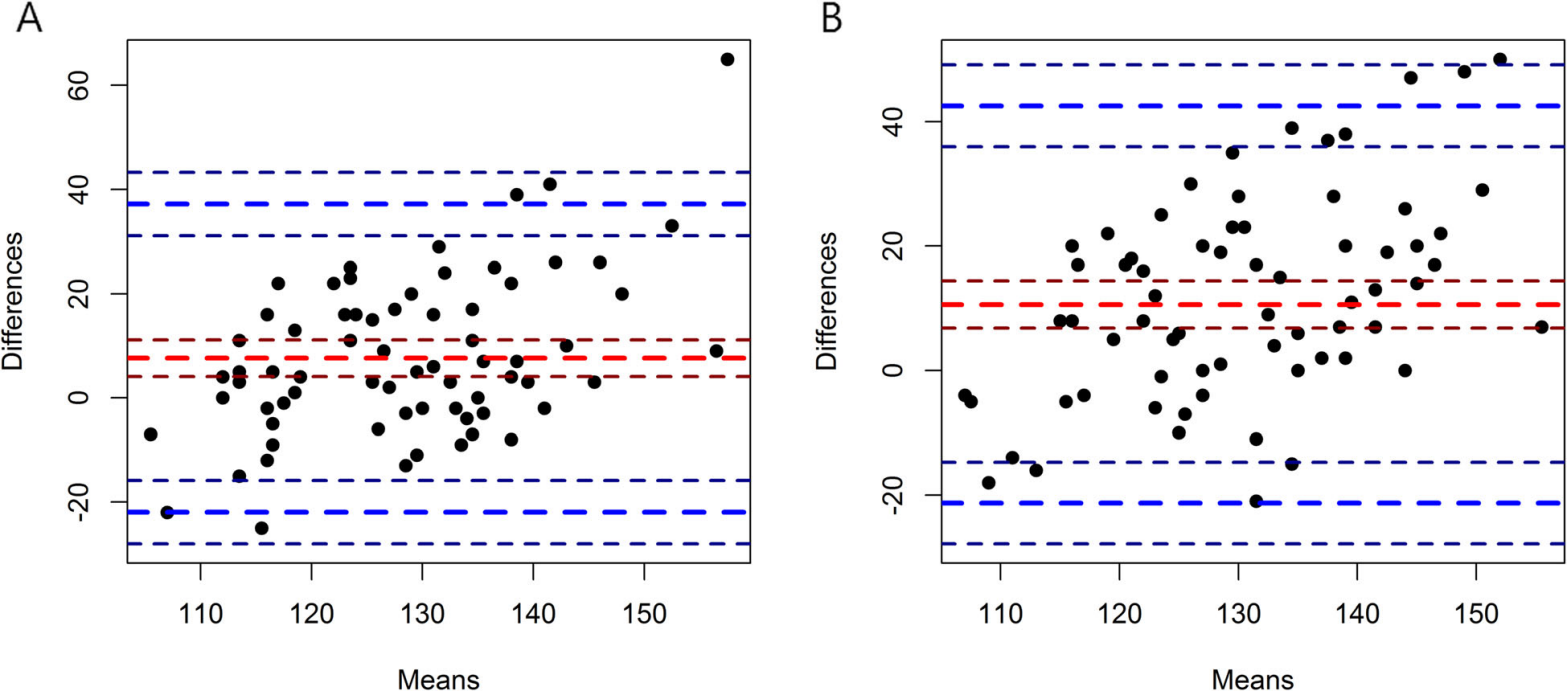
Bland-Altman plot comparing mean after-consult systolic blood pressure (**A**), before-consult systolic blood pressure (**B**) with 24 h ambulatory blood pressure (mmHg). AC; after consult, BC; before consult. Red dashed lines, mean bias; Blue dashed lines, 95 % limits of agreement

Overall blood pressure values are shown in Table 3. Significant difference was observed for the mean systolic BP values when BC BP and AC BP were performed. (135.4 ± 16.9 vs. 131.9 ± 16.4 mmHg, *P* = 0.015) (Fig. 2 A) However, there was no difference in the mean diastolic BP between BC BP and AC BP. (73.6 ± 11.8 vs. 74.4 ± 11.7 mmHg, *p* = 0.415) (Fig. 2B). Furthermore, systolic BC AOBP after see a doctor exhibited a significant difference when compared to systolic AC AOBP values. (125.1 ± 14.4 vs. 123.0 ± 14.1 mmHg, *p* = 0.006) (Fig. 2 A). In the diastolic AOBP values, there was also significant difference between BC AOBP and AC AOBP (72.0 ± 10.5 vs. 71.0 ± 9.8, mmHg *p* = 0.038) unlike BC and AC diastolic BP. (Fig. 2B) For clinical use of AC BP, we compared AC BP with BC AOBP. These difference were 6.33 ± 13.01 mmHg (*p* < 0.001), 2.14 ± 7.65 mmHg (*p* = 0.023) in systolic BP and diastolic BP, respectively (Fig. 3 A, 3B).

**Table 3:** Mean blood pressure values

Variables	BC BP	AC BP	BC AOBP	AC AOBP	24 h ABP	*P*-value
SBP, mmHg	135.37 ± 16.90	131.95 ± 16.40	125.17 ± 14.41	122.98 ± 14.09	124.54 ± 10.63	0.001
DBP, mmHg	73.75 ± 11.85	74.42 ± 11.71	71.99 ± 10.49	70.99 ± 9.83	75.17 ± 6.50	0.001
h, bpm			72.2 ± 11.0	70.7 ± 10.6		0.063

*BC* Before consult, *AC* After consult, *AOBP* Automated office blood pressure, *ABP* Ambulatory blood pressure, *SBP* Systolic blood pressure, *DBP* Diastolic blood pressure, *HR* Heart rate, *bpm* beat per minute

When compare among all BP values, both systolic and diastolic AC AOBP was lowest (*p* = 0.001) representing resting state.

Bland-Altman plots for the comparison of the systolic AC BP, BC BP and diastolic AC BP, BC BP with the systolic 24 h mean ABP are shown in Fig. 3 A, B, respectively. Differences between 24 h ABP and office BP were 10.61 mmHg for BC BP and 7.61 mmHg for AC BP, respectively. AC BP showed consistently narrower than BC BP.

For BP variability analysis, we checked difference between BC BP and AC BP and defined as delta office BP and analyzed correlation between delta office BP and 24 h BP deviation. There was no significant association between delta office BP and 24 h BP variability (*p* = 0.389).

**Fig. 4:**
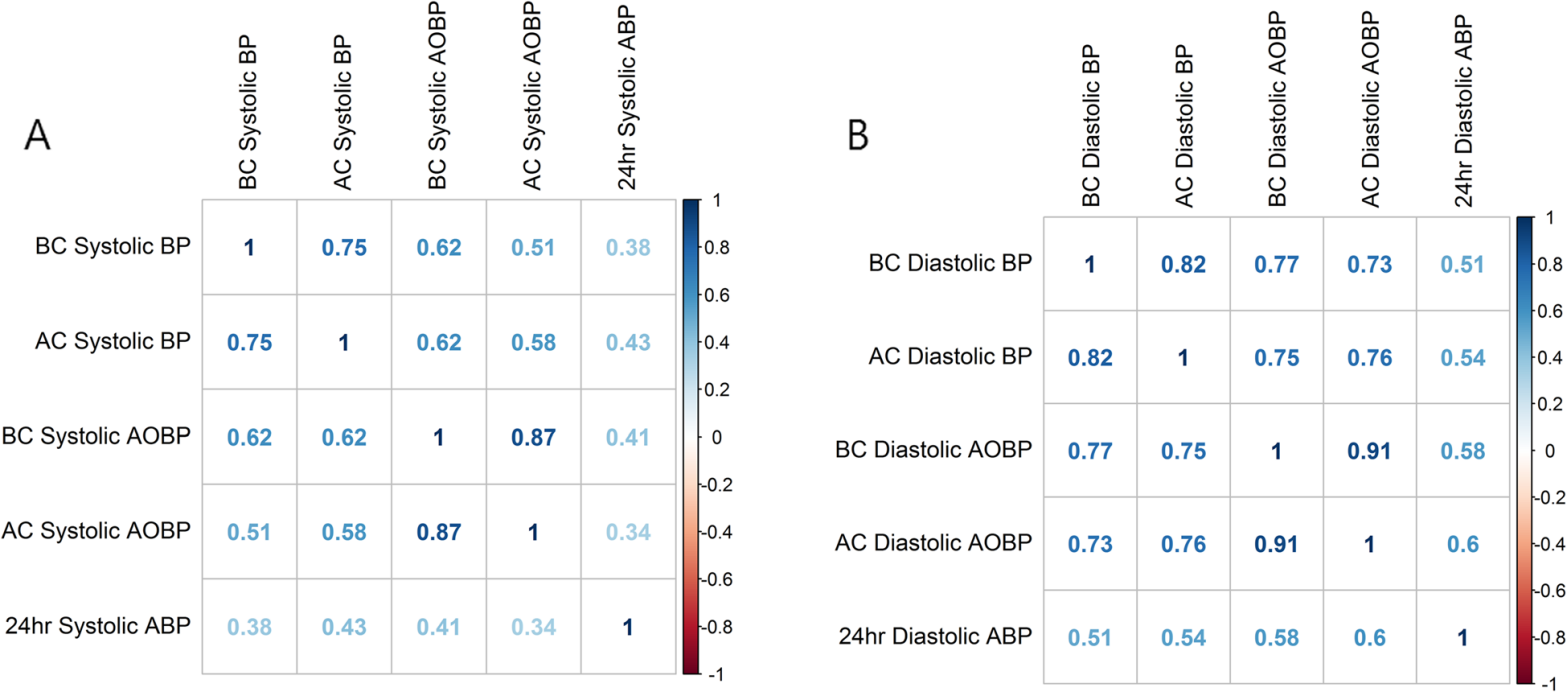
Correlation coefficient analysis among office blood pressure, automated blood pressure and 24 h ambulatory blood pressure. Systolic blood press (**A**), Diastolic blood pressure (**B**), BC; before consult, BP; blood pressure, AC; after consult, AOBP; automated office blood pressure, ABP; ambulatory blood pressure

For further evaluation, we analyzed correlation coefficient between 24 h ABP and BC BP, AC BP, BC AOBP, and AC AOBP. Their correlations are shown in Fig. 4 A, B. AC systolic BP (r = 0.43) was most correlated with 24 h ABP among 4 systolic BP measurements. For diastolic BP, AC AOBP (r = 0.6) was most correlated with 24 h ABP among 4 diastolic measurements.

We defined white coat effect as blood pressure difference > 20mmHg using before and after office BP. we compared clinical values including left ventricular hypertrophy, pulse wave velocity and Urine A/C ratio between patients with white coat effect and without white coat effect. However, there were no differences in the clinical values for assessing prognostic impact except urine A/C ratio (Table 4).

**Table 4:** Comparison clinical values between patients with white coat effect and without white coat effect

	White coat effect(+) *N* = 8	White coat effect(-) *N* = 74	*P* value
LVH	2 (28.57 %)	4 (4.76 %)	0.258
PWV, cm/sec	1547.17 ± 820.34	1607.58 ± 366.79	0.865
A/C ratio	12.66 ± 3.66	50.91 ± 103.90	0.007

*LVH* Left ventricular hypertrophy, *PWV* Pulse wave velocity, *A/C ratio* Urine albumin to creatinine ratio

Lastly, we compared BC and AC BP difference between men and women. There was no significant difference in BC SBP (133.4 ± 15.3 vs. 137.8 ± 18.6 mmHg, *p* = 0.269), AC SBP (129.2 ± 15.4 vs. 135.4 ± 17.1 mmHg, *p* = 0.101) and BC and AC BP differences (3.9 ± 12.0 vs. 2.2 ± 10.8 mmHg, *p* = 0.523) between men and women.

## Discussion

To the best of our knowledge, this is first study to simultaneously compare BP values between before and after seeing a doctor for reducing white coat effect and examined for any differences in the mean AOBP when these readings were obtained before and after office consult. We also compared the office BP and AOBP values with 24 h ABP, which is generally accepted as a more sensitive risk predictor than office BP of CV events, in order to investigate any differences between AOBP and 24 h ABP values [8, 9].

Our findings showed that based on the automated BP measurement device, AC systolic BP was lower when readings with after seeing a doctor were taken. Furthermore, AC systolic AOBP had further lower in the similar situation. The mild association between AC BP and 24 h ABP values was observed and the value was higher than other BP values. So, it should be highlighted that AC BP measurement should be considered as alternative routine practice.

The overall prevalence of white coat hypertension in the general population is estimated to be approximately 10–15 %, and it amounts to 30 % in patients with increased clinic BP recordings [10]. White coat hypertension is more frequent in women, non-smokers, and patients with low clinic BP and smaller left ventricular mass at echocardiography [11]. Although the prevalence of white coat effect in the hypertension treatment during follow up period is not exactly known, it might be similar with that of white-coat hypertension. In addition, it is hard to take a sufficient rest before BP measurement in South Korea because both patients and physicians are pressed for office consultation time.

In our study, white coat effect was about 9.8 % when we defined as 20mmHg difference between BC and AC systolic BP. So, AC BP that we measured BP after consult is fit to take a sufficient time and reduce white coat effect.

Recently, a study was performed for reducing white coat effect [12]. Emmanuel et al. examined the difference in AOBP readings, with and without 5 min of rest prior to three readings recorded at 1-min intervals. In that study, systolic AOBP can be initially checked without any preceding rest and if readings are normal can be accepted. However, when AOBP is ≥ 130 mm Hg, measurements should be rechecked with 5 min of rest. So, it has limitation for daily routine practice.

We still used 24 h ABP as reference standard. A recent meta-analysis showed that due to the significant heterogeneity it is believed that use of the AOBP should not replace daytime ABP.

Interestingly, it should be highlighted that AC systolic AOBP value was lower than 24 h ABP. Our findings are inconsistent with those of others [13, 14] that clinic BP values were higher than daytime ABP values in the higher range of BP distribution. We suggested even AOBP might have white coat effect and AC AOBP could reflect most comfortable resting BP state.

This study has several limitations. First, it should be mentioned that the entrance of the study personnel into the examination room before recording before or after-consult BP with 5 min resting cannot completely eliminate noisy circumstance. However, we believe that situation reflected more real clinical practice. Second, the relatively small size of the study population may limit generalization of study results. Third, we could not check the long-term prognosis because we concentrated on method of BP measurement. We will identify the adverse event in the near future.

## Conclusions

In our study, AC AOBP was most lowest representing resting state. Overall AC BP including routine BP and AOBP was lower than BC BP. Based on the present results, although AC BP was higher than BC AOBP, it might be used as alternative measurement in the routine clinical practice for reducing white coat effect.

## Data Availability

The datasets used and/or analyzed during the current study are available from the corresponding author on reasonable request.
